# Thermochromic microcapsules with highly transparent shells obtained through *in-situ* polymerization of urea formaldehyde around thermochromic cores for smart wood coatings

**DOI:** 10.1038/s41598-018-22445-z

**Published:** 2018-03-05

**Authors:** Xiaodong Zhu, Yu Liu, Zhao Li, Weicong Wang

**Affiliations:** 1 Key Laboratory of Bio-Based Material Science and Technology (Ministry of Education), Northeast Forestry University, Harbin, Heilongjiang 150040 China; 2 College of Materials and Engineering, Northeast Forestry University, Harbin, Heilongjiang 150040 China

## Abstract

In this paper, thermochromic microcapsules were synthesized *in situ* polymerization with urea formaldehyde as shell material and thermochromic compounds as core material. The effects of emulsifying agent and conditions on surface morphology and particle size of microcapsules were studied. It was found that the size and surface morphology of microcapsules were strongly depending on stirring rate and the ratio of core to shell. The stable and small size spherical microcapsules with excellent transparency can be obtained at an emulsifying agent to core to shell ratio as 1:5:7.5 under mechanical stirring at 12 krpm for 15 min. Finally, the thermochromic property was discussed by loading microcapsules in wood and wood coatings. Results indicate that microcapsules can realize the thermochromic property while incorporated with wood and coatings, and could have high potential in smart material fabrication.

## Introduction

The use of wood materials indoor helps to improve the energy efficiency of buildings, additionally, that can helps to improve human thermal comfort in physical and mental wellbeing^1^. With rapid development of novel materials, much attention is paid to functional and smart materials to improve the energy efficiency of building^2–5^. Thermochromic material is a kind of intelligent material that undergoes a series of color transitions with a specified temperature range. Introducing of thermochromic pigment into wood materials will help to improve the seasonal visual effect of wood. Moreover, it provides a new solution to energy consumption for interior buildings^6^.

Liu *et al*. first introduced thermochromic materials into wood products^7,8^. They colored poplar veneers by ultrasonic impregnation using crystal violet lactone, biphenyl A, 1-tetradecanol and sodium thiosulfate. Jiang et.al selected three kind of aliphatic alcohols as solvents to preparing thermochromic wood, they found the color-change temperature depended on the melting point of solvent^9,10^. Fu et.al improved the light fastness of thermochromic wood by adding ultraviolet absorber^11^. These studies adopted the impregnation treatment for thermochromic wood fabrication, which could cause a large consumption of raw materials, and most of the thermochromic agents contain phenols and alcohols, the unstable components can be affected by the environments and result in an adverse effect to environmental adaptability. Moreover, the loss of thermochromic compounds during wood fabrication would limit the scope of application.

Microencapsulation technology is an efficient method to solve these problems. Microcapsules have been in use in many fields, such as medicine, textile, food, adhesives, building concrete, etc.^12–18^, but only recently introduced in wood materials. Xu et.al prepared reversible thermochromic wood plastic composites with thermochromic microcapsules^19^. Hu et.al investigated the color-changing behavior of medium density fiberboard (MDF) by surface coating thermochromic microcapsules^20,21^. In the present studies, the thermochromic wood materials were fabricated incorporated with microcapsules coatings to achieve the thermochromic function. As is well known, the reactive and functional thermochromic core materials have been applied in some frontier fields, in which the functional microcapsules play an important role in thermochromic properties. However, the systematical investigation on microencapsulation of thermochromic pigment and the application in wood materials is still limited.

In this study, the thermochromic compound microcapsules were prepared via *in situ* polymerization method, and the optimal emulsifying conditions for preparing thermochromic microcapsules for application in wood materials were explored. In addition, the thermochromic performances of thermochromic microcapsules loading in wood and wood coatings were also discussed in this paper.

## Results and Discussion

### Determination of emulsifying agent

**Figure 1:**
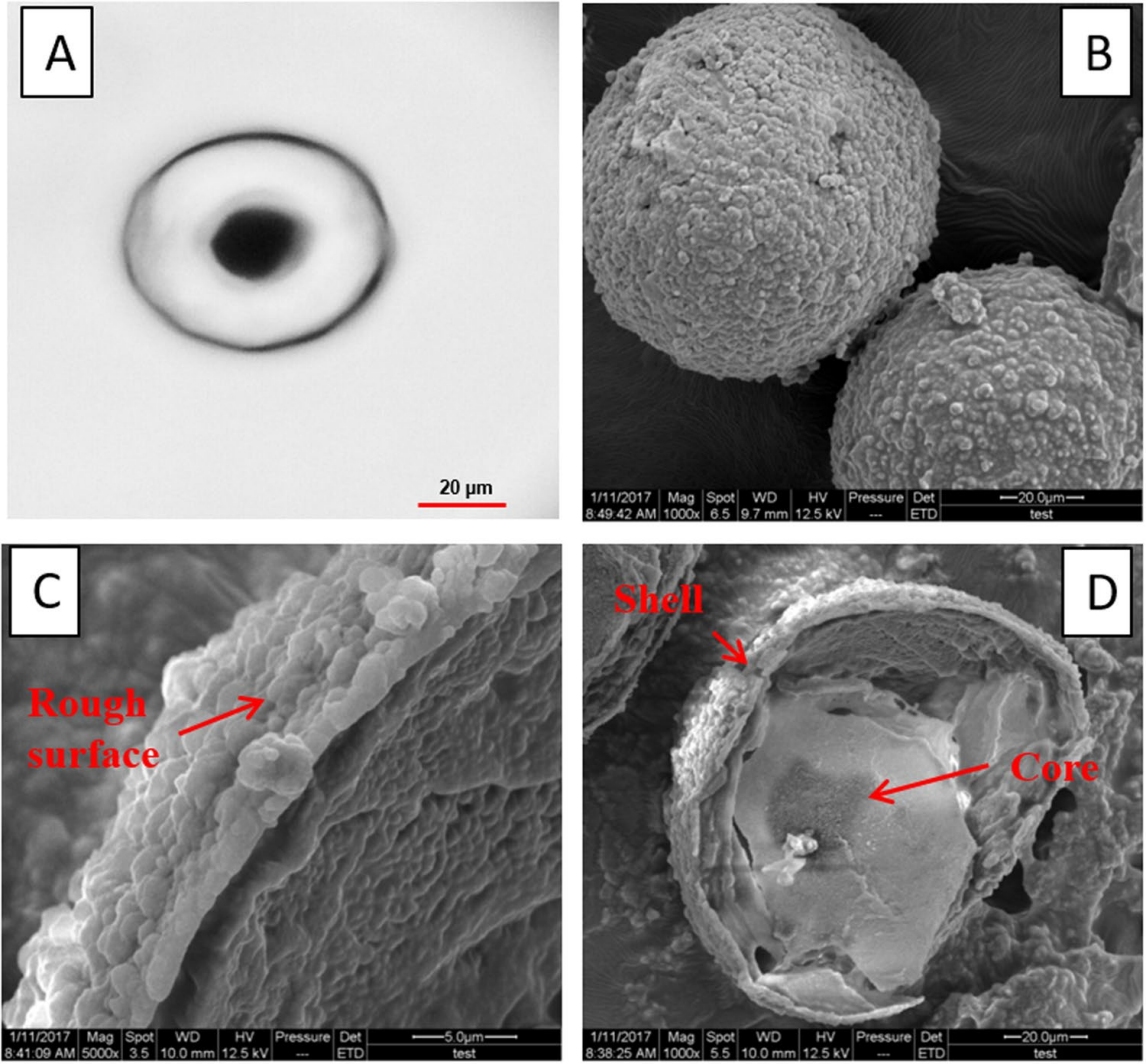
Optical (**A**) and SEM (**B**–**D**) images of microcapsules prepared with acacia emulsify agent.

Table 1 described the feature of five emulsification systems. The hydrophile-lipophile balance (HLB) values of sodium dodecyl benzene sulfonate (SDBS) and alkyl phenyl polyoxyethylene ether (OP-10) are lower than 1-tetradecanol, which is the solvent used for thermochromic compound synthesis, and they presented poor emulsification effect. The linkage of OP-10 ether bond and water molecules was unstable, as the temperature raised, the linkage was broken down and the hydrophilicity of OP-10 decreased, and the unstable emulsion system was formed. As an anionic emulsify agent, sodium dodecyl sulfate (SDS) presented strong emulsifying ability and it was beneficial to condensation polymerization of prepolymer on core material. However, a large number of bubbles generated during emulsification procedure, which had adverse effect on encapsulation and thermochromic property of core material. Polysorbate (Tween −80) presented good emulsification effect, but the poor affinity with UF prepolymer caused shell materials self- assembled, which prevented the encapsulation of thermochromic core materials and the formation of the microcapsules. Acacia presented intermediate emulsification effect. Acacia is composed of simple sugars galactose, arabinose, rhamnose, glucuronic acids and protein component^22^. The protein-rich high molecular mass component adsorbs preferentially onto the surface of the oil phase, and the carbohydrate blocks inhibit flocculation and coalescence through electrostatic and steric repulsions, which is beneficial to smooth and firm shell formation. On the other hand, as the acacia negative charged as the solution pH value greater than 2.2, it can adsorb the positive charged prepolymers to form the microcapsules. Figure 1 showed the morphology of microcapsules prepared with acacia emulsifying agent.

**Table 1:** The feature of emulsification system and thermochromic microcapsules prepared by different emulsify agents.

Emulsify agent	Features description
Acacia	Intermediate emulsification effect, a small number of microcapsules with large particle size formed, core materials maintain thermochromic property
SDBS	Poor emulsification effect, unstable emulsion system
OP-10	Poor emulsification effect, unstable emulsion system
Tween-80	Good emulsification effect, few microcapsules formed
SDS	Excellent emulsification effect, a lot of microcapsules formed, serious adhesion phenomenon, ash green appearance, core materials loss thermochromic property

**Figure 2:**
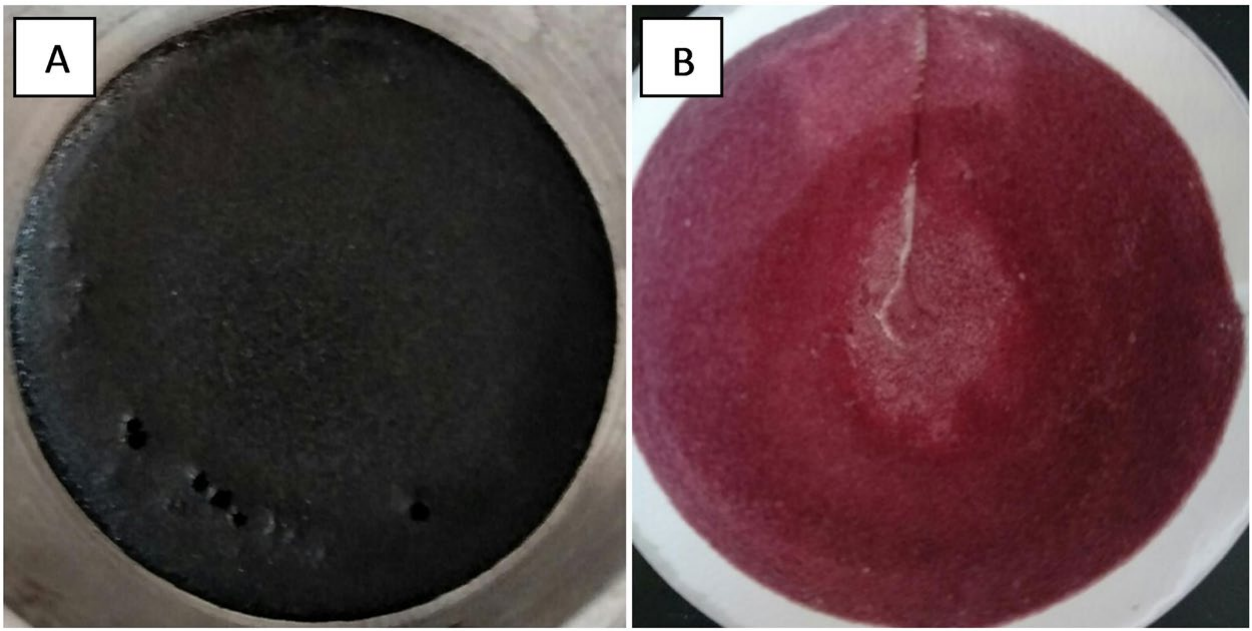
Images of thermochromic compounds before (**A**) and after (**B**) microencapsulation.

The stability of core droplets in emulsifying system affects the microcapsule morphology and particle size during the encapsulation procedure. As Fig. 1 showed, the microcapsule and core material presented spherical shape, which indicated good dispersion and emulsifying effect of acacia. The magnified SEM of thermochromic microcapsules displayed the surface of UF shell material and the broken shell- core structure. The UF prepolymer was dissolved in water and then polycondensation reaction formed UF polymers as the pH value of solution adjusted to acid^23,24^. The reaction of UF prepolymer at the thermochromic compounds interface formed the capsule shell, and the surface of microcapsules gradually became coarse and it was covered with granular deposits as the reaction going on. The rough surface resulted from the deposition and agglomeration of UF polymer. The shell wall of microcapsule was transparent, and it was beneficial to thermochromic property. It was also observed that the color of microcapsules was different from thermochromic compounds. The encapsulation of thermochromic compounds caused light refraction of UF shell and showed bright color in visible light (Fig. 2).

### Effect of fabrication conditions on microcapsules

The prerequisite for manufacturing thermochromic microcapsules with uniformed size, smooth non-porous wall surface morphology and thermochromic properties is related to the emulsion conditions^25,26^. The effects of stirring rate, emulsifying time, ratio of emulsifying agent to core and ratio of core to shell on microcapsule particle size and morphology were investigated in this study. Table 2 showed the orthogonal experiment results of thermochromic microcapsules.

**Table 2:** Orthogonal experiment results.

Tests	Factors				Average particle size/μm
	Ratio of emulsifying agent to core (A)	Stirring rate (B)/rpm	Emulsifying time (C)/min	Ratio of core to shell (D)	
1	1(1:6)	1(8)	1(5)	1(1:1.5)	56.33
2	1(1:6)	2(10)	2(15)	2(1:1.75)	64.49
3	1(1:6)	3(12)	3(25)	3(1:2)	52.98
4	2(1:5)	1(8)	2(15)	3(1:2)	51.85
5	2(1:5)	2(10)	3(25)	1(1:1.5)	54.37
6	2(1:5)	3(12)	1(5)	2(1:1.75)	57.21
7	3(1:4)	1(8)	3(25)	2(1:1.75)	67.67
8	3(1:4)	2(10)	1(5)	3(1:2)	53.33
9	3(1:4)	3(12)	2(15)	1(1:1.5)	43.46

**Figure 3:**
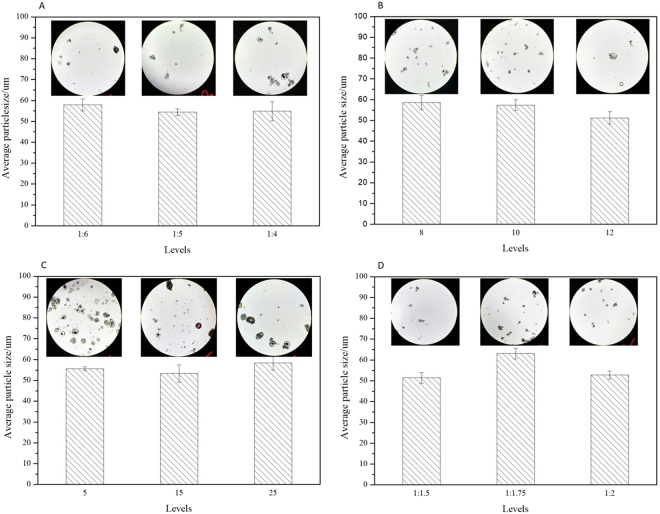
Range analysis of four factors to average thermochromic particle size.

Range analysis and ANOVA analysis were used to determine the optimal conditions and evaluate the significance of emulsifying factors on mean particles size at α = 0.05 level (Table 3 and Fig. 3). It indicated that stirring rate and the ratio of core to shell had significant effect on average particle size of thermochromic microcapsules.

**Table 3:** ANOVA results for average thermochromic particle size.

Source	Sum of Squares	df	Mean Square	F	Sig.
A	43.519	2	21.759	0.997	0.406
B	188.882	2	94.441	4.327	0.048
C	77.346	2	38.673	1.772	0.224
D	495.513	2	247.756	11.352	0.003
Error	196.433	9	21.826		
Total	56933.437	18

Previous work showed that the size control of microcapsules could be realized in three stages, emulsification, pH adjustment and curing^27^. Range analysis of fabrication conditions on the microcapsules size and standard errors were shown in Fig. 3. As illustrated in Fig. 3A and Table 3, the emulsifying agent dosage showed no significant effect on mean microcapsule size. The microcapsules prepared with emulsifying agent to core ratio as 1:5 showed the smallest mean particle size as 54.48 μm. The small dosage of emulsifying agent (as the ratio of emulsify agent to core was 1:6) led to the insufficient dispersion of mixture and large micelle particle size. Meanwhile, large amount of emulsifying agent (as the ratio of emulsify agent to core was 1:4) increased the viscosity of reaction mixture, which suppressed the redispersion of emulsion droplet and resulted in a small microcapsule size distribution.

Figure 3B presented the trend of microcapsule size changed with stirring rate. When the stirring rated increased, small size of microcapsules were obtained. Since high speed of emulsification increased the shear stress to the droplet, the droplet would be dispersed to smaller diameter; also the higher stirring rate could reduce the aggregates of UF prepolymer curing and deposited on core surface, which helps to form the thermochromic microcapsules with small particle sizes. With shown in Fig. 3C, the microcapsule size changed with emulsifying time. The microcapsules can be any shape and resulted in a wide particle size distribution as the emulsifying time of 5 min. The increase of emulsifying time causes the small core- shell size and distribution are uniform. It can be seen that multi-core microcapsules were observed as the emulsifying time increased to 25 min (Fig. 3). This phenomenon can be attributed to the good dispersion of core material, the small and uniform core materials encapsulated by UF polymer and the excess UF prepolymer cured on the microcapsules surface. Therefore, the large size and rough multi-core microcapsules were formed.

**Figure 4:**
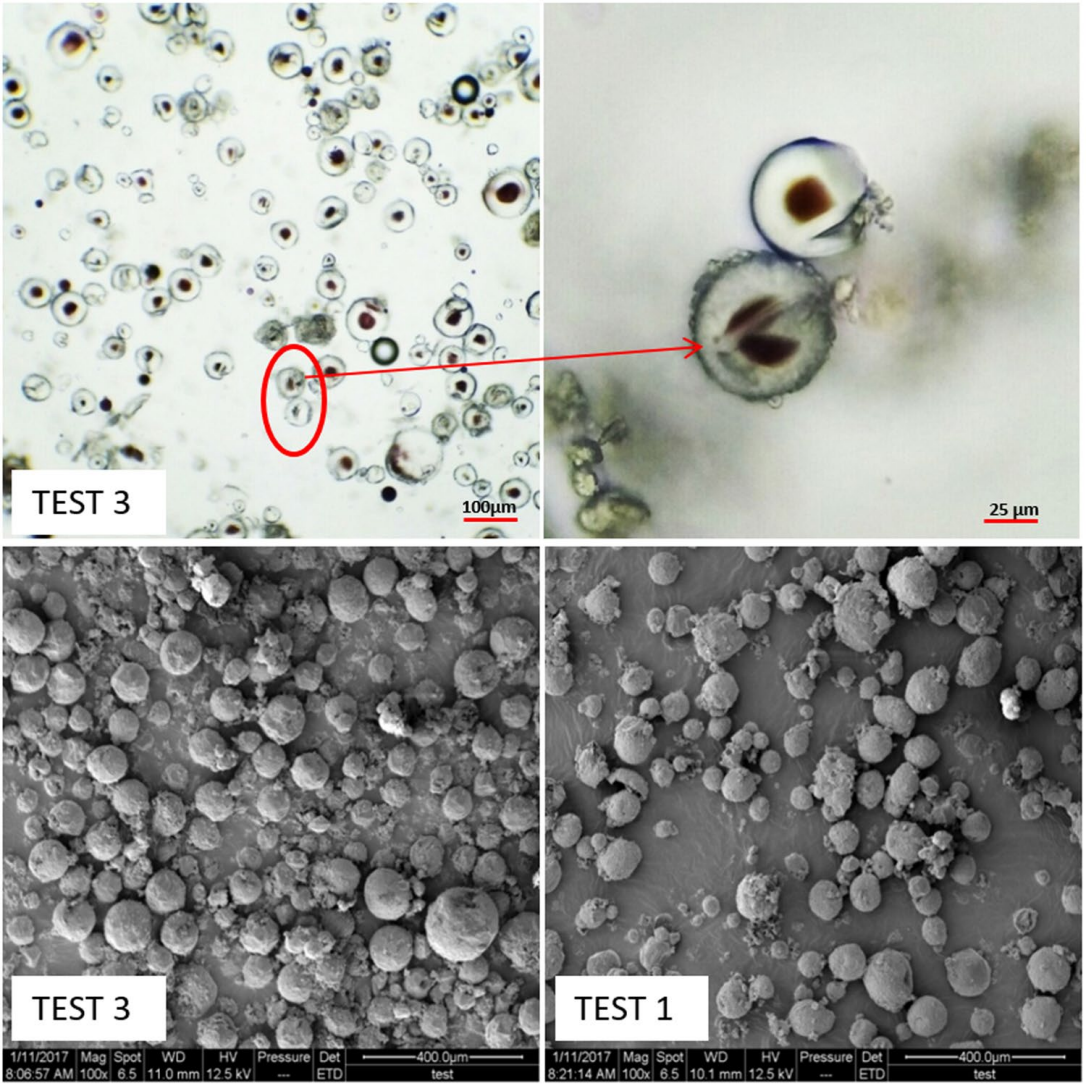
Optical and SEM images of multi-core microcapsules.

For low ratio of shell wall materials, little amount of UF prepolymer was cured and deposited on the core surface and could not form a stable core- shell structure. When the ratio of core to shell increased, numerous colloidal UF polymer particles enriched and deposited on the micelle surface, and the stable microcapsules with large size were obtained. Excessive cured UF polymer shell resulted in the roughness morphology surface, as Fig. 4 shows. The curing and deposition of UF polymer strengthened the mechanical properties of shell wall, but the optical performance became worse.

To obtain the stable and optimal surface morphology microcapsules with minimum size, the microencapsulation of thermochromic compounds could carried out at an emulsifying agent to core to shell ratio as 1:5:7.5 under mechanical stirring at 12 krpm for 15 min.

### Properties of thermochromic wood materials

**Figure 5:**
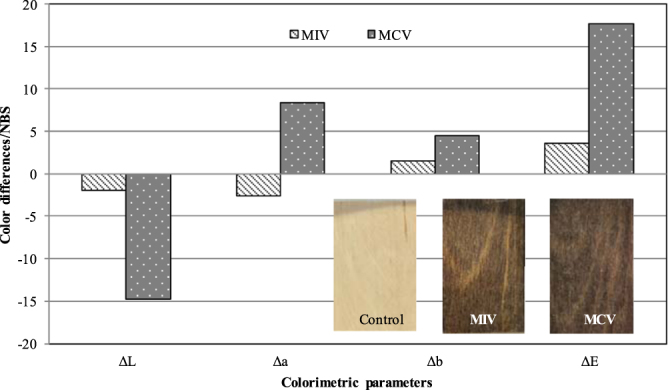
Color differences values of MIV and MCV.

The incorporation of microcapsules into a wood varnish system is a simple mechanical stirring work. In order to compare the effect of varnish on thermochromic properties of microcapsules, colorimetric parameters of wood veneers treated with thermochromic microcapsules aqueous solution were also recorded. Figure 5 shown the color differences values of wood veneers treated with microcapsules aqueous solution (MIV) and microcapsules varnish coatings (MCV). Compared with color change values during 0 °C to 70 °C, MCV showed higher ΔE values. It was found that MIV and MCV displayed brown hue, lightness of all thermochromic wood veneers decreased, and the color of MCV was darker than MIV. The decrease of lightness was attributed to the increase of coating thickness and the decrease of surface roughness, which resulted in the reduction of diffuse reflection on the surface^28^. Comparison of color characteristics between control veneer, MIV and MCV, the redness and greenness chromaticity of MIV declined, and the blueness and yellowness chromaticity of MIV and MCV goes up. It indicated that the MIV exhibited greenish and yellowish color, whereas the MCV exhibited reddish and yellowish color than control wood veneers. This was associated with the waterborne varnish, which contains red and yellow impurities.

**Figure 6:**
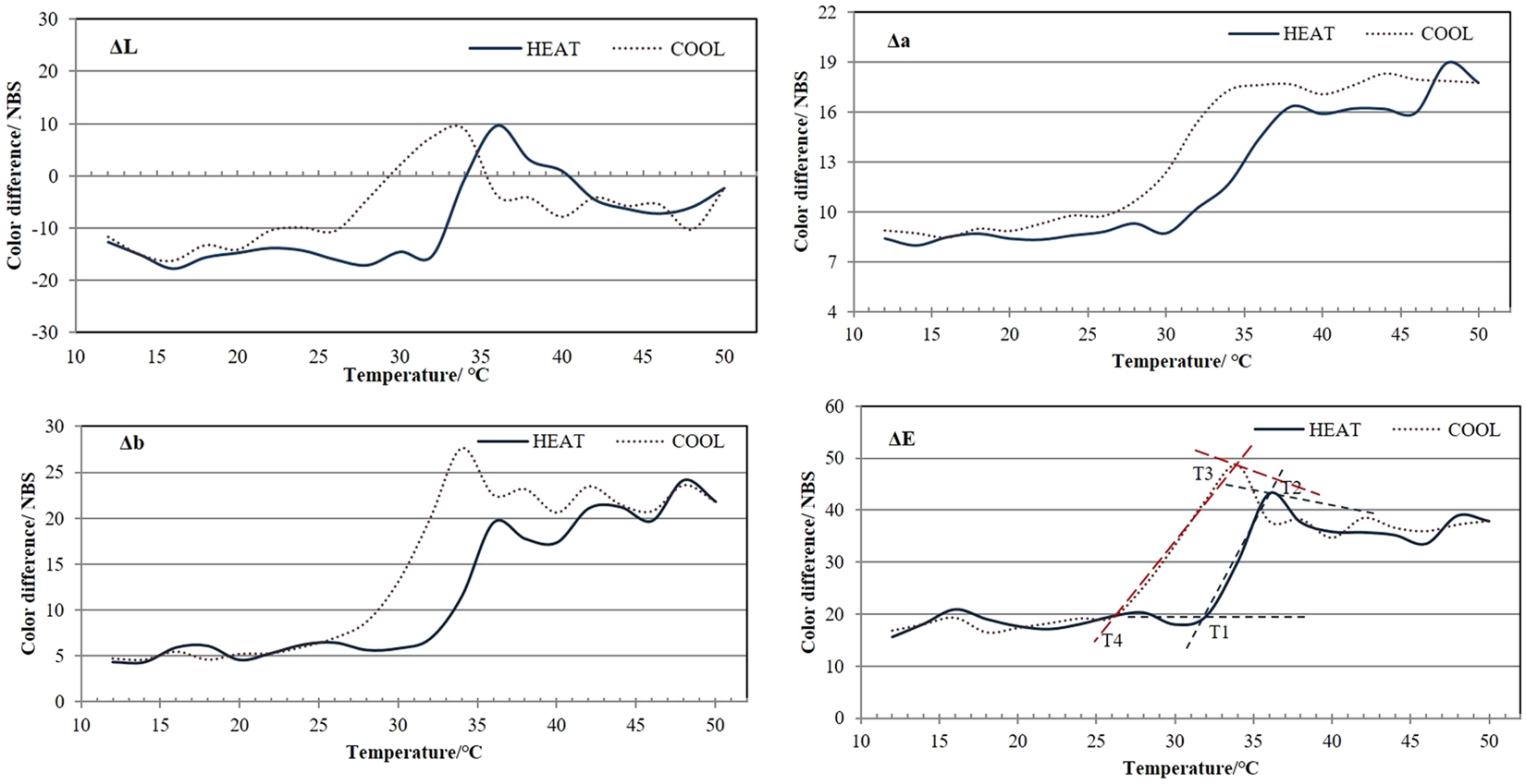
Color difference values of thermochromic microcapsules coating veneers during heat and cool process. T1 and T2 are the initial and final achromatic temperature, while T3 and T4 are initial and final chromatic temperature.

The color characteristics of thermochromic wood materials were depended on temperature and procedure is illustrated in Fig. 6. T1 and T2 described the initial and final achromatic temperature during decolourization procedure. As the temperature below 31 °C, the color parameters (L, a, b) were rarely change, and the color change value ΔE tended to be stable. Between 31–37 °C decolourization occurs. Combined with Fig. 6, the color parameters (L, a, b) increased significantly. As the temperature above 37 °C, the decolourizations slow down. It is known from the literature that reversible thermochromic change occurs via two competing reactions^29^. At low temperature, the solvent exists in its solid form in leuco dye- developer- solvent system, as the temperature increased, the solvent melts, the leuco dye- developer system convert to colorless state.

During the chromatic procedure, as the temperature above 34 °C, the color parameters a and b rarely changed, the ΔL and ΔE values slightly increased with the temperature ranged from 36–34 °C. T3 and T4 describe the initial and final chromatic temperature during reverse action. As the temperature ranged between 34–26 °C, the color parameters significantly reduced and the ΔE values enlarged, the system regains color. Based on the color change characteristic, it can be deduced that the initial and final chromic temperature were 34–26 °C during reverse action. Perfect reversible process should return to the same color after cooling. It can be seen from the graphic, the color hysteresis phenomenon occurs between heated and cooled state. This phenomenon was also found in previous studies^30^.

**Figure 7:**
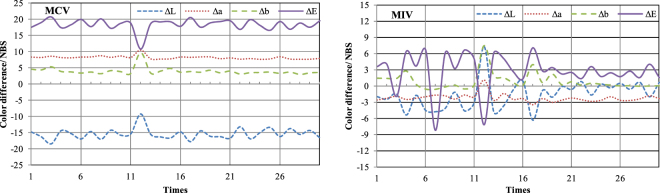
Curves of color change during 30 times heat- cool loops.

The reversible stability of thermochromic property was evaluated according to the change of colorimetric parameters in heat- cool loops. Figure 7 showed the colorimetric parameters values of MIV and MCV in 30 times heat-cool loops.

It can be seen from Fig. 7, for MCV samples, Δa values were rarely changed after 30 times heat-cool loops, the Δb, ΔL and ΔE values were slightly fluctuated during the circles. However, the color differences values of MIV showed dramatic fluctuation during 30 times heat-cool loops, this might associate with the unstable connection between microcapsules and wood, and the loss of the efficiency of thermochromic microcapsules during the heat- cool loops.

## Conclusion

Microcapsulation technology has a good potential use in textile, film, fluorescence indication, and electronics^31–33^. The purpose of this study was to investigate the thermochromic properties of wood materials fabricated with thermochromic microcapsules. The thermochromic core material was the mixture of ODB-2, bisphenol- A and 1- tetradecanol, and shell wall was urea formaldehyde resin. The thermochromic compounds could be encapsulate by UF polymer using *in-situ* polymerization. Microcapsules prepared in the existence of acacia emulsifying agent presented stable and excellent spherical shape, and their core- shell structure was verified by SEM. The optimal thermochromic microcapsules can be prepared at an emulsifying agent to core to shell ratio as 1:5:7.5 under mechanical stirring at 12 krpm for 15 min. Finally, wood veneers were treated by the thermochromic microcapsules. All of the wood veneers samples treated with microcapsules aqueous solution and microcapsules coatings exhibited good thermochromic properties. The results of color differences confirmed that thermochromic microcapsules decreased the lightness of treated wood, blueness and yellowness chromaticity went up and the ΔE values changed remarkably. The color changed within a temperature range of 31–37 °C during heat procedure, and the reverse action occurs within the temperature range of 34–26 °C. Color hysteresis was found during heat and cool circles. It was also found that thermochromic wood coatings had good thermal stability. Therefore, it would be a great potential to be applied in wood materials.

## Materials and Methods

### Materials

Poplar veneers, with an area of 150 × 110 mm and 2.82 mm thickness, were supplied by Taoshan Corporation (Harbin, China), the average moisture content and density were 7% and 0.395 g/cm^3^.

2-anilino-6-(dibutylamino)−3-methyl fluoran (ODB-2, purity 99.9%), bisphenol A (purity 99.9%) and 1-tetradecanol (purity 99.9%) were used for thermochromic compounds synthesis, and they were supplied by Jingchun chemicals co., Ltd (Shanghai, China).

Analytical reagent urea (purity 99.9%) and formaldehyde (purity 37.0–40.0%) were supplied by Tianli chemical reagents Ltd (Tianjin, China). Chemical grade gum arabic, SDBS, OP-10, SDS and Tween 80 were bought from Kemiou chemical reagent Co. Ltd (Tianjin, China). Citric acid, sodium hydroxide and sodium chloride are chemical grade and supplied by Hengxing chemical reagent Ltd (Tianjin, China). Waterborne polyurethane varnish was supplied by North paint, China.

### Methods

#### Preparation of thermochromic compounds

**Figure 8:**
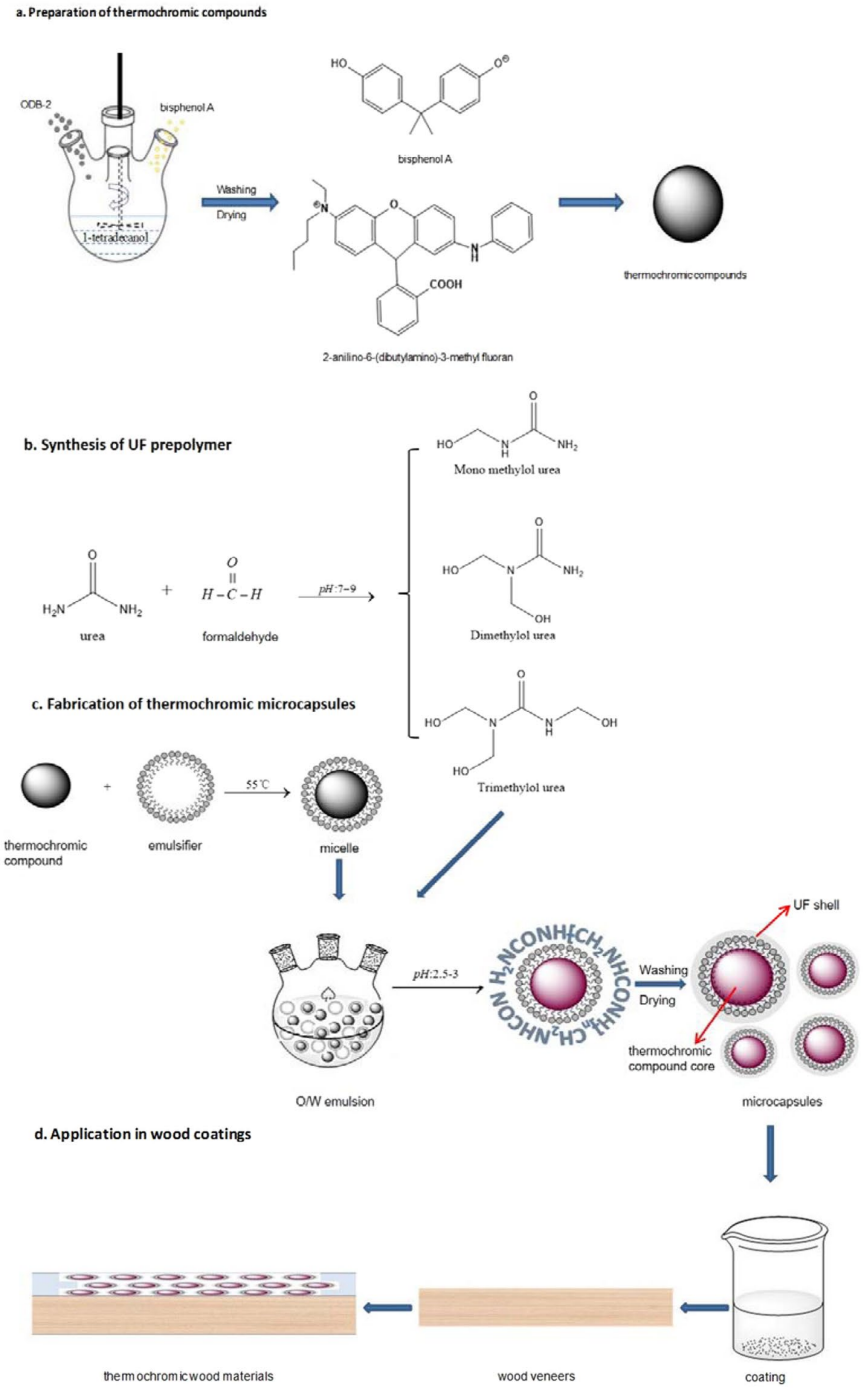
Schematic diagram for thermochromic microcapsules fabrication and their application in wood veneers.

Thermochromic compounds were synthesized by mixing ODB-2, bisphenol A and 1-tetradecanol in the ratio of 1:2:60 and heated in boiling flask-4-neck at 70 °C water bath. The mix components were stirred with a teflon paddle at 600 rpm for 1 hour. The reversible thermochromic compounds were obtained after natural cooling. The experimental process was shown in Fig. 8a.

#### Synthesis of urea formaldehyde (UF) prepolymer

A certain amount of aqueous formaldehyde solution and urea were added into a boiling flask-3-neck in the molar ratio of 1.7:1. The pH of the solution was adjusted to 7–9 with 2.5 w.t.% NaOH solution. The mixture was heated to 75 °C in 35 min. After 1 hour reaction and stirred at 600 rpm, the UF prepolymer was obtained (Fig. 8b).

#### Fabrication of thermochromic microcapsules

The thermochromic microcapsules were prepared using *in situ* polymerization procedure. A certain amount of thermochromic compounds were melted and mixed with emulsifying agent solution in 55 °C water bath under homogenization shearing. Gum arabic, sodium dodecylbenzenesulphonate, alkyl phenyl polyoxyethylene ether, polyoxyethylenesorbitan monooleate and sodium lauryl sulfate were investigated as the emulsifying agents to achieve the optimal emulsify effect. Through a vigorous agitation, the stable micelles were obtained. The UF prepolymer was added into the oil micelles mixture at 35 °C, which consist of emulsifying agents and thermochromic compounds, and stirred at 600 rpm. The pH value of O/W emulsion was kept between 2.5 to 3 by adding citric acid for 1 h. After pH adjustment, a few drops of sodium chloride solution were added into the mixture and stirred at 400 rpm. The temperature of water bath was slowly raised to 65 °C and maintained for 40 min to complete the reaction. The synthesized microcapsule suspension was cooled down, filtered and air dried (Fig. 8c).

#### Fabrication of thermochromic wood materials

Thermochromic wood materials were fabricated by surface finishing with microcapsule varnish. The thermochromic microcapsules were added into the waterborne varnish in the ratio of 20 w.t.% and stirred at 500 rpm for 20 min. The wood veneers were finished with one layer of primer and two layers of top coat. 80 g per m^2^ weights of modified varnish was applied on the wood surface with short-haired brush. The films were left for 7 days at room temperature for complete curing.

### Experiment design

#### Emulsifying agent

Emulsification is the action of a liquid dispersed in an insoluble liquid with a tiny droplet, which is an interfacial phenomenon occurred between two insoluble liquids. The emulsifier is a surfactant with hydrophilic and lipophilic groups, which formed an adsorption layer on the interface to reduce the oil- water phase interfacial tension^34^. The appropriate emulsifier plays an important role to obtain well-dispersed core materials and stable emulsion. The hydrophilic- lipophilic balance (HLB) value is an important parameter for choosing emulsify agent. For a system using surfactants with HLB values between 8 and 16, O/W emulsification is obtained. Table 4 presented the HLB values of emulsifying agents used in this study^35^.

**Table 4:** HLB values of commonly used O/W emulsifying agent.

Trade name	Chemical name	HLB value
Acacia	Gum arabic	8.0
SDBS	Sodium dodecylbenzenesulphonate	10.638
OP-10	Alkyl phenyl polyoxyethylene ether	14.5
Tween-80	Polyoxyethylenesorbitan monooleate	15.0
SDS	Sodium lauryl sulfate	40.0

The dosage of emulsifying agent was 15.0 w.t.% of core materials, they were melt in a certain amount of deionized water and mixed with thermochromic core materials in 55 °C water bath under homogenization shearing of 3000 rpm for 5 min. After a few minutes standing, UF prepolymer was added for microcapsules preparation. The appropriate emulsifying agent was determined according to optical microscope and SEM analysis of microcapsules morphology.

#### Orthogonal experiments design of fabrication conditions

Microcapsules fabrication conditions affect the sizes of thermochromic particles and the stability in continuous phase. In this study, orthogonal experiment design was adopted to select the optimal condition for microcapsules fabrication. Table 5 lists the factors and levels. The average particle size was calculated to evaluate the emulsification degree.

**Table 5:** Factors and levels.

Levels	Factors			
	A Ratio of emulsifying agent to core	B Stirring rate/krpm	C Emulsifying time/min	D Ratio of core to shell
1	1:6	8	5	1:1.5
2	1:5	10	15	1:1.75
3	1:4	12	25	1:2

### Property measurements and analysis

#### Characterization of microcapsules

The morphology of microcapsules was analyzed using KSZ-4GA optical microscopy (Xiwan, China) at 40× and 100× magnifications. Three hundred particles were selected randomly from optical microscopic photographs and their diameters were measured to calculate the average diameters. Scanning electron microscopy (SEM) (JSM-7500F, Japan) was used to examine the external morphology of microcapsules at 12.5 kV.

#### Analysis of thermochromic properties

The thermochromic veneer samples were conditioned in consistent cabinet at 50% RH to equilibrium at the temperature set at 0–70 °C. The colorimetric parameters of wood surface were measured according to CIElab system. The average color parameters, including L (lighness index), a (red-green index) and b (yellow-blue index) of wood veneer surface measured by NP10QC chroma meter (3NH, Inc., China).

The color difference value ΔE was calculated with the following equation:

1 $${\rm{\Delta }}E=\sqrt{{\rm{\Delta }}{L}^{2}+{\rm{\Delta }}{a}^{2}+{\rm{\Delta }}{b}^{2}}$$

## Electronic supplementary material

Supplementary dataset
